# Correction: Phonetic differences between affirmative and feedback head nods in German Sign Language (DGS): A pose estimation study

**DOI:** 10.1371/journal.pone.0321229

**Published:** 2025-03-27

**Authors:** Anastasia Bauer, Anna Kuder, Marc Schulder, Job Schepens

The Funding statement for this paper is incorrect. The correct Funding statement is as follows: This work is supported as a short-term collaboration grant between Anastasia Bauer, Anna Kuder and Marc Schulder supported by the Programme DFG SPP 2392 Visual Communication (ViCom), Frankfurt am Main, Germany. Anna Kuder is funded by a DFG grant awarded to Anastasia Bauer [project number 502013233] within the DFG Priority Programme 2392 Visual Communication (ViCom, 2022-2025).

Anastasia Bauer and Job Schepens are supported by the Cluster Development Program “Language Challenges” funded by the University of Cologne. Job Schepens is additionally funded by the DFG Collaborative Research Centre (CRC 1252) “Prominence in Language” [project number 281511265] based at the University of Cologne.

Marc Schulder’s contribution to the publication has been produced in the context of the joint research funding of the German Federal Government and Federal States in the Academies’ Programme, with funding from the Federal Ministry of Education and Research and the Free and Hanseatic City of Hamburg. The Academies’ Programme is coordinated by the Union of the Academies of Sciences and Humanities.

The funders have no role in study design, data collection and analysis, decision to publish, or preparation of the manuscript.
