# Supplementary material for: Phonetic differences between affirmative and feedback head nods in German Sign Language (DGS): A pose estimation study
Source: PLoS One. 2024 May 30;19(5):e0304040. doi: 10.1371/journal.pone.0304040 (PMC11139280; doi:10.1371/journal.pone.0304040)
Supplement: S1 File — (PDF) [file pone.0304040.s001.pdf]

The annotations and code produced in the context of this article are publicly available at https:doi.org/10.5281/zenodo.10838847. The archive contains the following:

- the manual annotations created for the article in ELAN (.eaf) format;
- the Python code used for quantitative analysis of the annotations with the aid of OpenPose data from MY DGS – annotated;
- the R code used to perform the statistical analyses discussed in the article as well as the depicted graphs.
